# Association of dietary calcium intake at dinner versus breakfast with cardiovascular disease in U.S. adults: the national health and nutrition examination survey, 2003–2018

**DOI:** 10.1186/s12889-024-18587-7

**Published:** 2024-04-19

**Authors:** Ting Zhang, Sijia Zhuang, Yue Yu, Zizhuo Fan, Xiyun Ren

**Affiliations:** 1 Clinical Medicine Specialty, the First Affiliated Hospital of Harbin Medical University, 150081 Harbin, Heilongjiang Province P. R. China; 2 Preventive Medicine Specialty, College of Public Health, Harbin Medical University, 150081 Harbin, Heilongjiang Province P. R. China; 3 Experimental Center for Preventive Medicine Teaching, College of Public Health, Harbin Medical University, 150081 Harbin, Heilongjiang Province P. R. China

**Keywords:** Dietary calcium, Cardiovascular disease, National health and nutrition examination survey, Dietary substitution model, Breakfast and dinner

## Abstract

**Background:**

Currently, it is still largely unknown whether the proportion of calcium intake at breakfast and dinner is associated with cardiovascular disease (CVD) in the general population.

**Objectives:**

The aim of this study was to evaluate the association of dietary calcium intake at dinner versus breakfast with CVD in a nationally representative sample of US adults.

**Methods:**

The study population consisted of 36,164 US adults (including 4,040 CVD cases) from the NHANES 2003 to 2018. According to the ratio of dietary calcium intake at dinner and breakfast (Δ = dinner/breakfast), 36,164 participants were divided into five groups. After adjustment for a series of confounder factors, logistic regression analyses were performed to examine the association between Δ and CVD. Dietary substitution models were used to explore the changes in CVD risk when a 5% dietary calcium intake at dinner was substituted with dietary calcium intake at breakfast.

**Results:**

Compared with participants in the lowest quintile, participants in the highest quintile were more likely to have CVD, with an adjusted OR of CVD of 1.16 (95% CI, 1.03 to 1.31). When the total calcium intake remained constant, replacing a 5% dietary calcium intake at dinner with dietary calcium intake at breakfast was associated with a 6% lower risk of CVD.

**Conclusions:**

Compared to the lowest quintile of Δ, participants in the highest quintile of Δ were likely to experience CVD in the general population. It is necessary to scientifically allocate dietary calcium intake at breakfast and dinner.

## Introduction

Cardiovascular disease (CVD), a group of heart and blood vessel diseases, includes myocardial infarction, angina pectoris, heart failure, heart attack, and stroke, and is the leading cause of death worldwide, with 17.5 million related deaths annually (31% of global deaths) [1]. In the U.S., CVD is the most common chronic disease, affecting 48% of adults [2]. Therefore, CVD has become a significant public health problem that contributes to the global burden of morbidity and mortality.

Dietary factors, as an important regulatory factor, play a critical role in the prevention and management of CVD. Calcium, in particular, is an essential constant element in the human body. It is not only very important for maintaining bone health but also plays an important role in cardiovascular health, as calcium can regulate blood vessels, muscle contraction, nerve conduction, hormone secretion, etc [3]. Meanwhile, dietary calcium can improve blood lipids, fat mass, and blood pressure, which are key risk factors for CVD [4, 5].

Circadian rhythms are biological rhythms with a period of approximately 24 h that allow organisms to prepare for the daily fluctuations brought on by day–night cycles, aligning internal biological functions with environmental changes. In mammals, circadian rhythms are regulated by circadian clocks. Previous studies have demonstrated that many cardiovascular functions, such as endothelial function, thrombus formation, blood pressure, and heart rate, are now known to be regulated by the circadian clock [6]. Diet is one of the most important external factors affecting the synchronization of these endogenous biological clocks [7]. Nutrient intake and its timing may play an important role in circadian rhythm mechanisms. In recent years, an increasing amount of evidence has shown that calcium can modulate physiological oscillations in circadian pacemaker neurons, and reset the clock gene expression cycle [8]. Currently, the effects between dietary factors (including nutrient and intake time) and circadian rhythmicity are clear in this new research area called “chrononutrition” [9]. However, it is still largely unknown whether calcium intake at different times over a whole day is associated with the CVD in the general population.

To examine this question, this study evaluated the association of calcium intake at dinner versus breakfast with the risk of CVD using data from the National Health and Nutrition Examination Survey (NHANES).

## Methods

### Study design and participants

The NHANES is a cross-sectional and nationally representative survey of the non-institutionalized civilian population in the U.S., with data released in two-year cycles to evaluate the health and nutritional status of the population and track changes therein over time. Participants were first interviewed at home to collect background information and then visited a mobile testing center to provide other relevant data, including anthropometry, blood pressure and laboratory measurements [10]. Detailed information on the NHANES has been provided previously [11]. Data accumulation was performed by the National Center for Health Statistics with approval from their ethics review board.

A total of 76,848 participants in the NHANES from 2003 to 2018 were enrolled in this study. Excluded participants included those under 20 years old (*n* = 29,085), pregnant women (*n* = 853), participants with missing essential information on dietary calcium intake at breakfast or dinner (*n* = 10,154), and participants with a total energy intake <500 kcal/day or > 4,500 kcal/day or using calcium supplements (*n* = 592). After the exclusion of such cases, the total number of subjects for this study was 36,164 adults (17,456 men and 18,708 women).

### Dietary survey

Dietary intake was measured with a 24-hour dietary recall completed on two nonconsecutive days. The first 24-hour dietary recall was conducted in person, and the second 24-hour dietary recall was conducted 3–10 days afterward via telephone. Dietary nutrients were estimated by using the guidelines of the U.S. Department of Agriculture’s Food and Nutrient Database for Dietary Studies. Dietary data were based on the mean of two dietary surveys. In the NHANES, mealtimes were divided into six parts: breakfast, morning snack, lunch, afternoon snack, dinner, and evening snack. For each participant, the amount of each food intake was listed according to mealtimes. Dietary measurements included dietary calcium in each meal (mg/day), total calcium(mg/day), energy (kcal/day), total dietary fat (g/day), protein (g/day), carbohydrate (g/day), saturated fatty acid (SFA, g/day), monounsaturated fatty acid (MUFA, g/day) and polyunsaturated fatty acids (PUFA, g/day). The detailed process of dietary data measurement is described on the NHANES database’s official website (https://www.cdc.gov/nchs/nhanes/ContinuousNhanes/Default.aspx).

### Main exposure and outcome measure

Dietary calcium intake was assessed via two dietary recalls of all types and amounts of food consumed at breakfast and dinner. In order to better control the variability of calcium intake between individuals at breakfast and dinner and better reflect the metabolic balance, the exposure variable of this study was the proportion of dietary calcium intake between dinner and breakfast (Δ = dinner/breakfast). The outcome of interest was CVD, defined as the self-reported diagnosis history of heart failure, coronary heart disease, angina/angina pectoris, heart attack, or stroke [12].

### Confounding measurements

The confounding covariates included age (years), gender (men/women), education level (less than 9th grade, 9-11th grade, high school graduate, GED or equivalent, some college or associate’s degree, or college graduate or above), smoking status (never smoked, current smoker, former smoker), moderate physical activity (yes/no), marital status (married, widowed, divorced, separated, never married), annual household income (≤$20,000, >$20,000), drinking (drinks/week), high-density lipoprotein cholesterol (HDL-c, mmol/L), total cholesterol (TC, mmol/L), uric acid (UA, mg/dL), hypertension status (yes/no), type 2 diabetes mellitus (T2DM) status (yes/no), and body mass index (BMI, kg/m^2^). Definitions of never smoked, current smoker, and former smokers were adopted from a previous study [13]. The amount of alcohol consumed was measured by number of drinks, wherein a standard drink was any drink that contained about 0.6 fluid ounces or 14 g of pure alcohol [14]. T2DM was defined by a self-reported diagnosis, an HbA1c level ≥6.5%, or a fasting plasma glucose level ≥7.0mmol/L. Hypertension was defined as persistent systolic blood pressure measurements of ≥ 140 mmHg and/or ≥ 90 mmHg of diastolic blood pressure. BMI was calculated as weight in kilograms divided by the square of height in meters.

### Statistical analysis

All statistical analyses were performed using R software version 3.5.3 (www.r-project.org/). The proportion of dietary calcium intake between dinner and breakfast (Δ) was categorized into quintiles. General linear models and chi-square tests were used to compare baseline characteristics by quintiles. Continuous variables were expressed as mean ± standard deviation and classified variables were expressed as percentages. A *P*-value of less than 0.05 (2-sided) was considered statistically significant. Missing covariables of less than 5% were filled in by multiple interpolation. When the missing value of a variable was greater than 5%, it would be deleted to avoid affecting the results.

### Logistic regression models

Logistics regression models were used to analyze and evaluate Δ and the risk of CVD in different models, and odds ratios (ORs) and their 95% confidence intervals (CIs) were estimated by three different logistics regression models with the lowest quintile of Δ as the reference category. Model 1 was adjusted for age, gender, education level, household income, smoking status, drinking status, physical activity and marital status. Based on the model 1 covariates, model 2 was further adjusted for total calcium, energy, carbohydrate, protein, fat, SFA, MUFA, PUFA, TC, HDL-c and serum UA levels. Finally, model 3 was further adjusted for hypertension status, T2DM status and BMI.

### Dietary substitution models

Dietary substitution model is a new statistical method that holds total dietary calcium intake constant to evaluate changes in the risk of CVD with a theoretical shift of the dietary calcium at breakfast and dinner. The method of substitution analysis is used to study the substitution of dietary calcium at dinner with dietary calcium at breakfast under the premise of equal calcium intake, and then observed changes in epidemiological indicators. We established an equivalent dietary calcium substitution model to evaluate the change in CVD risk caused by switching dietary calcium intake from a single time period to another single time period. This study performed a series of dietary substitution models to assess changes in the risk of CVD when a 5% dietary calcium intake at dinner was substituted with a 5% dietary calcium intake at breakfast, according to the different definition modes of breakfast and dinner.

### Sensitivity analysis

In the NHANES, mealtimes were divided into six parts: breakfast, morning snack, lunch, afternoon snack, dinner, and evening snack. Moreover, the timing of breakfast and dinner intake was defined differently in different studies. Therefore, this study performed four sets of sensitivity analyses to verify the stability of the relationship between Δ and the risk of CVD. First, breakfast and a morning snack were considered breakfast, and the study data were reanalyzed. Second, dinner and an evening snack were considered dinner. Third, when breakfast and a morning snack were considered breakfast and dinner and evening snack as dinner, the study data were analyzed again. The fourth sensitivity analysis was performed on overweight/obesity people.

## Results

### Baseline characteristics

A total sample of 36,164 people was used in this study, including 4,040 CVD cases. The characteristics of the baseline population in terms of Δ in quintiles are shown in Table 1. Age, gender, smoking, education level, physical activity, total calcium, energy, protein, carbohydrate, fat, total SFA, MUFA, PUFA intake, serum UA level, T2DM status, hypertension status, and BMI were significantly different across quintiles 1–5 (*p* < 0.05). There was no significant difference in drinking status, serum TC, and HDL-c levels among quintiles (*P* > 0.05).

**Table 1:** Characteristics of the participants according to the ratio of calcium at dinner versus breakfast in NHANES (2003–2018)

	Q1 (*N* = 7,233)	Q2 (*N* = 7,233)	Q3 (*N* = 7,232)	Q4 (*N* = 7,233)	Q5 (*N* = 7,233)	*p* value
Age (years)	51.1(19.2)	51.1(19.2)	50.6(19.0)	48.2(18.6)	45.2(17.6)	< 0.001
Gender (men) (n, %)	3,462(47.9)	3,434(47.5)	3,419(47.3)	3,455(47.8)	3,686(51.0)	< 0.001
Education (College graduate or above) (n, %)	1,397(19.3)	1,782(24.6)	1,877(26.0)	1,843(25.5)	1,609(22.2)	< 0.001
Married (n, %)	3,641(50.3)	3,830(53.0)	3,895(53.9)	3,914(54.1)	3,602(50.0)	< 0.001
Current Smoking (n, %)	1,201(16.7)	1,180(16.3)	1,262(17.5)	1,427(19.7)	1,939(26.8)	< 0.001
Drinking (drinks/week) Moderate physical activity (n, %)	4.1(37.1) 2,802(38.7)	3.8(35.2) 3,046(42.1)	3.1(22.4) 3,135(43.3)	3.0(19.1) 3,094(42.8)	3.9(31.6) 3,154(43.6)	0.291 < 0.001
Income (>$20,000)	5,214(72.1)	5,428(75.0)	5,469(75.6)	5,598(77.4)	5,501(76.1)	< 0.001
Total energy (kcal/d)	1,917.9(728.9)	2,013.1(747.5)	2,034.3(745.7)	2,079.3(763.1)	2,077.3(783.3)	< 0.001
Total protein (g/d)	77.0(33.0)	80.3(33.1)	80.3(32.9)	81.4(33.6)	79.8(33.8)	< 0.001
Total carbohydrates (g/d)	240.7(95.6)	248.9(98.3)	249.0(98.8)	249.3(99.9)	246.3(102.3)	< 0.002
Total fat (g/d)	70.0(34.1)	75.2(35.1)	77.2(35.0)	80.8(36.3)	81.3(36.6)	< 0.001
Total SFA (g/d)	22.5(12.0)	24.2(12.5)	25.0(12.4)	26.4(13.2)	26.6(13.1)	< 0.001
Total MUFA (g/d)	25.2(13.2)	27.1(13.5)	27.8(13.4)	29.0(13.9)	29.3(13.9)	< 0.001
Total PUFA (g/d)	15.9(8.9)	17.2(9.2)	17.6(9.3)	18.2(9.3)	18.2(9.7)	< 0.001
Total calcium (mg/d)	929(487.5)	920.4(458.9)	916.5(466.2)	903.7(473.6)	856.2(458.7	< 0.001
UA (mg/dL)	5.4(1.5)	5.4(1.4)	5.4(1.4)	5.4(1.4)	5.4(1.4)	< 0.001
TC (mmol/L)	4.9(1.1)	4.9(1.1)	4.9(1.1)	4.9(1.1)	4.9(1.1)	0.130
HDL-c (mmol/L)	1.4(0.4)	1.4(0.4)	1.4(0.4)	1.4(0.4)	1.4(0.4)	0.529
Hypertension (n, %)	1,465(20.2)	1,423(20.0)	1,442(19.9)	1,324(18.3)	1,217(16.8)	< 0.001
T2DM (n, %)	1,308(18.1)	1,302(18.0)	1,245(17.2)	1,150(15.9)	922(12.7)	< 0.001
BMI (kg/m^2^)	28.7(6.4)	28.8(6.7)	28.9(6.8)	29.1(7.1)	29.3(7.3)	< 0.001

Continuous variables are expressed as mean (SD); Categorical variables are expressed as *N* (%); Generalized linear models and χ2 test were used to probe for differences in continuous variables and categorical variables; Q, quintile. SFA, saturated fatty acid. MUFA, monounsaturated fatty acid. PUFA, polyunsaturated fatty acid. HDL-c, high-density lipoprotein cholesterol. TC, total cholesterol. UA, uric acid. T2DM, type 2 diabetes mellitus. BMI, body mass index

### Logistic regression models

The logistic regression results of the relationship between Δ and the risk of CVD in the whole population are showed in Table 2. The results of research demonstrated that compared with the lowest quintile, the highest quintile of Δ was more likely to increase the risk of CVD. The adjusted OR of CVD was 1.16 (95% CI, 1.03 to 1.31) after controlling for age, gender, education level, household income, smoking status, drinking status, physical activity, marital status, total calcium, energy, carbohydrate, protein, fat, SFA, MUFA, PUFA, TC, HDL-c, serum UA level, hypertension status, T2DM status and BMI.

**Table 2:** The relationship between the risk of CVD and the ratio of dietary calcium intake at dinner versus breakfast by logistic regression models (*N* = 36,164)

	Q1	Q2	Q3	Q4	Q5	*p* for trend
Ratio	< 0.35	0.35–0.92	0.92–2.09	2.09–6.56	≥ 6.56	
Case/*N*	863/7,233	900/7,233	876/7,232	744/7,233	657/7,233	
Model1	1(Ref.)	1.09(0.98,1.22)	1.11(1.00,1.24)	1.07(0.95,1.20)	1.15(1.03,1.30)	0.045
Model2	1(Ref.)	1.10(0.98,1.23)	1.12(1.00,1.26)	1.07(0.95,1.20)	1.15(1.02,1.30)	0.059
Model3	1(Ref.)	1.09(0.98,1.22)	1.13(1.00,1.26)	1.07(0.95,1.20)	1.16(1.03,1.31)	0.044

Model 1 was adjusted by age, gender, education level, household income, smoking status, drinking status, physical activity, and marital status; Model 2 was further adjusted by for total calcium, energy, carbohydrate, protein, fat, SFA, MUFA, PUFA, TC, HDL-c, and serum UA levels; Model 3 was further adjusted for hypertension status, T2DM status and BMI. Case/*N*, number of case subjects/total; Q, quintile SFA, saturated fatty acid. MUFA, monounsaturated fatty acid. PUFA, polyunsaturated fatty acid. HDL-c, high-density lipoprotein cholesterol. TC, total cholesterol. UA, uric acid. T2DM, type 2 diabetes mellitus. BMI, body mass index

### Dietary substitution analysis

**Fig. 1:**
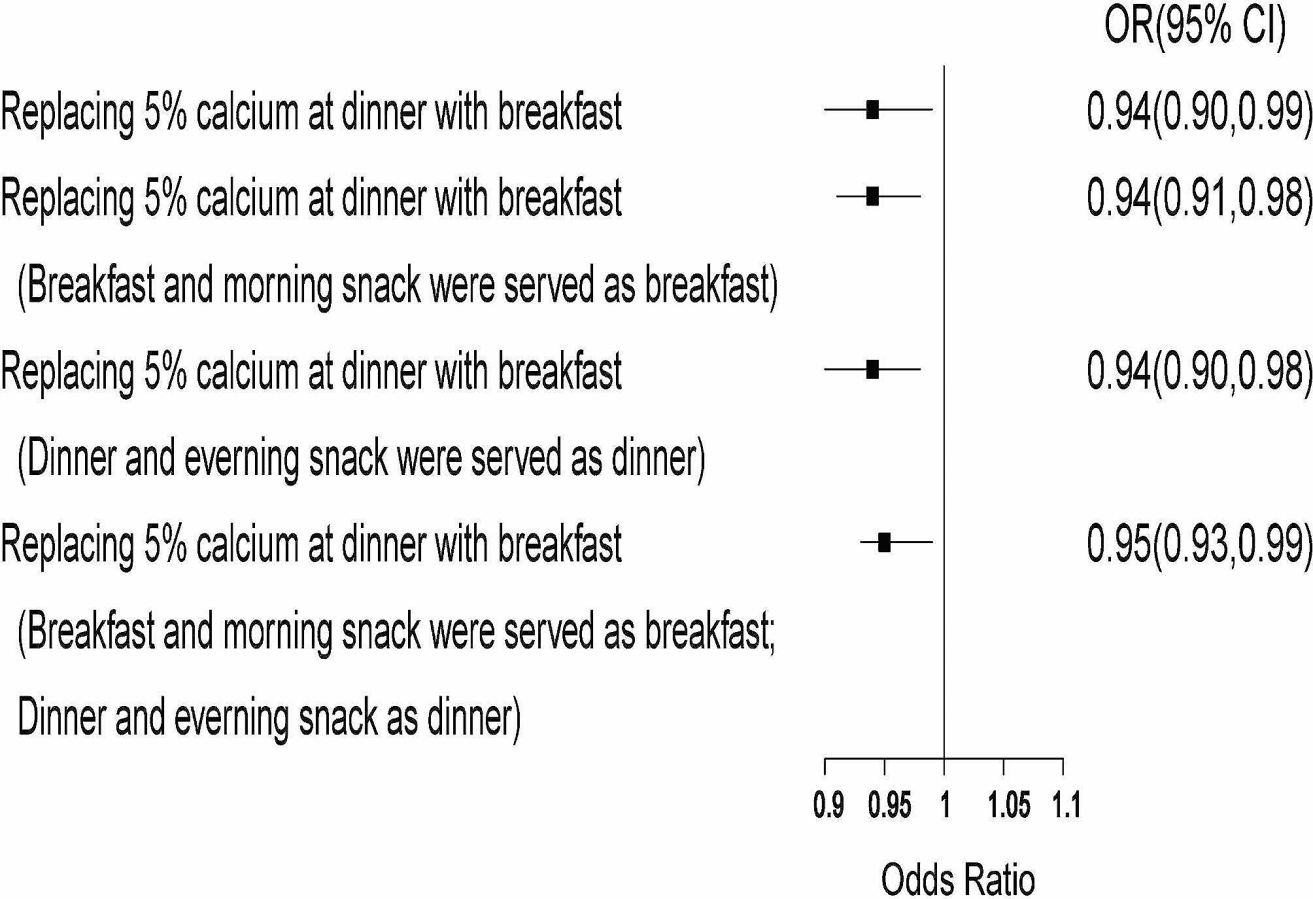
Multivariate adjusted ORs for CVD: equivalent substitution of dietary fiber from breakfast and lunch. Adjusted ORs for CVD risk: Dietary substitution of calcium intake from dinner to breakfast. Adjustments included age, gender, education level, household income, smoking status, drinking status, physical activity, marital status, total calcium, energy, carbohydrate, protein, fat, SFA, MUFA, PUFA, TC, HDL-c, serum UA level, hypertension status, T2DM status and BMI

Four sets of dietary substitution analyses are shown in Fig. 1, in which the changes of CVD risk were demonstrated. The result of research showed that when 5% of dietary calcium consumed at dinner were replaced by 5% of dietary calcium consumed at breakfast, the CVD risk decreased by 6% (OR: 0.94, 95% CI 0.90 to 0.99). When breakfast and a morning snack were considered as breakfast, or dinner and an evening snack were considered as dinner, the CVD risk also decreased by 6% (OR: 0.94, 95% CI 0.91 to 0.98; OR: 0.94, 95% CI 0.90 to 0.98). When breakfast and a morning snack were consumed as breakfast, and dinner and an evening snack were consumed as dinner, the CVD risk decreased by 5% (OR: 0.95, 95% CI 0.93 to 0.99).

### Sensitivity analysis

According to different definitions of breakfast and dinner, three sets of sensitivity analyses were performed, and the results were consistent with previously reported outcomes shown in Table 3. Compared with the lowest quintile, when breakfast and a morning snack were consumed as breakfast, or dinner and an evening snack were consumed as dinner, the highest quintile of Δ indicated a significant decrease in the risk of CVD, and the adjusted OR were 1.14 (95% CI, 1.01 to 1.29) and 1.14 (95% CI, 1.01 to 1.29), respectively. When dinner and an evening snack were consumed as dinner and breakfast and a morning snack were consumed as breakfast, the adjusted OR was also 1.14 (95% CI, 1.01 to 1.29). Meanwhile, in the overweight/obesity population, compared with the lowest quintile, the adjusted OR of CVD in the highest quintile of Δ was 1.18 (95% CI, 1.03 to 1.36) after controlling for a series of confounding factors.

**Table 3:** Four sensitivity analysis results for the relationship between the risk of CVD and the ratio of dietary calcium intake at dinner versus breakfast according to different definitions of breakfast and dinner (*N* = 36,164) and in overweight/obesity population by logistic regression models (*N* = 25,224)

	Q1	Q2	Q3	Q4	Q5	*p* for trend
**Sensitivity analysis 1**						
Ratio	< 0.34	0.34–0.87	0.87–1.98	1.98–5.89	≥5.89	
Case/*N*	863/7,233	900/7,233	876/7,232	744/7,233	657/7,233	
Model1	1(Ref.)	1.08(0.97,1.20)	1.12(1.00,1.25)	1.05(0.94,1.18)	1.14(1.01,1.28)	0.081
Model2	1(Ref.)	1.08(1.97,1.21)	1.12(1.00,1.26)	1.05(0.94,1.18)	1.13(1.01,1.27)	0.110
Model3A	1(Ref.)	1.08(0.96,1.21)	1.12(1.00,1.26)	1.05(0.93,1.18)	1.14(1.01,1.29)	0.091
**Sensitivity analysis 2**						
Ratio	< 0.40	0.4–0.98	0.98–2.24	2.24–6.93	≥6.93	
Case/*N*	876/7,233	898/7,233	862/7,232	746/7,233	658/7,233	
Model1	1(Ref.)	1.06(0.95,1.18)	1.07(0.96,1.19)	1.06(0.95,1.19)	1.14(1.02,1.29)	0.047
Model2	1(Ref.)	1.07(0.96,1.20)	1.08(0.96,1.20)	1.06(0.95,1.20)	1.14(1.01,1.28)	0.069
Model3A	1(Ref.)	1.06(0.95,1.19)	1.07(0.95,1.20)	1.06(0.94,1.19)	1.14(1.01,1.29)	0.061
**Sensitivity analysis 3**						
Ratio	< 0.39	0.39–0.95	0.95–2.12	2.12–6.20	≥6.20	
Case/*N*	872/7,233	895/7,233	865/7,232	750/7,233	658/7,233	
Model1	1(Ref.)	1.07(0.96,1.19)	1.08(0.97,1.20)	1.06(0.94,1.18)	1.14(1.02,1.28)	0.049
Model2	1(Ref.)	1.07(0.96,1.20)	1.09(0.97,1.22)	1.05(0.94,1.19)	1.14(1.01,1.28)	0.069
Model3A	1(Ref.)	1.07(0.95,1.19)	1.08(0.97,1.21)	1.05(0.93,1.18)	1.14(1.01,1.29)	0.059
**Sensitivity analysis 4**						
Ratio	< 0.35	0.36–0.91	0.91–2.07	2.07–6.55	≥ 6.55	
Case/*N*	664/5,045	695/5,044	679/5,045	592/5,044	521/5,045	
Model1	1(Ref.)	1.08(0.96,1.23)	1.14(0.98,1.26)	1.08(0.95,1.23)	1.17(1.02,1.34)	0.040
Model2	1(Ref.)	1.09(0.96,1.24)	1.13(0.99,1.28)	1.08(0.95,1.24)	1.16(1.01,1.33)	0.071
Model3B	1(Ref.)	1.09(0.96,1.24)	1.13(0.99,1.29)	1.09(0.95,1.25)	1.18(1.03,1.36)	0.035

Model 1 was adjusted by age, gender, education level, household income, smoking status, drinking status, physical activity, and marital status; Model 2 was further adjusted by for total calcium, energy, carbohydrate, protein, fat, SFA, MUFA, PUFA, TC, HDL-c, and serum UA levels.; Model 3 A was further adjusted for hypertension status, T2DM status and BMI. Model 3B was further adjusted for hypertension status and T2DM status based on model 2. Case/*N*, number of case subjects/total; Q, quintile SFA, saturated fatty acid. MUFA, monounsaturated fatty acid. PUFA, polyunsaturated fatty acid. HDL-c, high-density lipoprotein cholesterol. TC, total cholesterol. UA, uric acid. T2DM, type 2 diabetes mellitus. BMI, body mass index.

## Discussion

To the best of our knowledge, this was the first study to discuss the association between Δ and the risk of CVD using a large population-based study of U.S. adults. The study observed a positive association between Δ and CVD risk after adjusting for some potential confounders. Moreover, substituting 5% of dietary calcium intake at dinner with the same intake at breakfast decreased the risk of CVD by 6%, emphasizing the importance of dietary calcium intake distribution across breakfast and dinner. In particular, it is necessary to reduce dietary calcium intake at dinner.

Currently, the evidence for the relationship between dietary calcium intake and CVD risk is insufficient and controversial. Studies have shown that too much or too little calcium intake has adverse effects on CVD [15, 16]. It has also been reported that calcium intake at the upper tolerable levels (2000–2500 mg/day) is not associated with CVD risk in generally healthy adults [17]. Conversely, epidemiological investigations show that increasing dietary calcium intake plays a positive role in the prevention and treatment of CVD [17–19]. It can be seen that the difference in the total calcium intake dosage may lead to the difference in results. Interestingly, previous studies have only investigated the relationship between the total calcium intake and the risk of CVD without considering the time dimension.

In recent years, some studies have demonstrated that the circadian clock system can interact with nutrients to influence bodily function [20]. In mammals, circadian oscillations in physiology and behavior are controlled by a master clock located in the suprachiasmatic nucleus of the hypothalamus [21]. Circadian rhythms have been extensively studied in the cardiovascular system. For example, Calvo Fernández Jr. et al. found that circadian rhythm disturbances contribute to CVD [22]. Particularly, patients with sleep disorders and shift workers have an increased risk of CVD [6]. CVD has always been a major problem that needs to be solved urgently across the world.

A previous study has demonstrated that the circadian clock regulates many cardiovascular functions, such as endothelial function, thrombus formation, blood pressure, and heart rate [6], and that a circadian rhythm disorder contributes to increased CVD risk. Studies have further found that mammals have a circadian rhythm for their dietary calcium absorption and metabolism [23] and that calcium can reset the clock gene expression cycle [8]. Bmal1, one of the core clock genes, controls circadian rhythms and regulates a very large diversity of physiological processes. The peak and valley times of the Bmal1 protein are located at zeitgeber time (ZT)6 and ZT18, respectively [24]. Cardiac mitochondria exhibit a higher calcium retention capacity and higher rates of calcium uptake during the sleep period, which is associated with a higher expression of the clock gene Bmal1.

In addition, circadian rhythm can also regulate the inflammatory NFκB pathway [25] and regulate an organism’s metabolism and immune system to adapt to seasonal and environmental changes [26]. A study has shown that a low Bmal1 expression decreased the gene expression of pro-inflammatory cytokines and increased the gene expression of antioxidative and anti-inflammatory factors. As is well-known, inflammation can increase a greater risk of atherosclerosis and insulin resistance, which are the leading mechanisms in the development of CVD. Therefore, low dietary calcium intake at breakfast may also promote inflammatory responses through the regulation of circadian rhythm genes, resulting in adverse cardiovascular consequences. Based on these findings, the timing of dietary calcium intake is very important for cardiovascular risk.

The alteration of stress vulnerability could be another mechanism to explain our observations. Animal studies show that during sleep, retaining mitochondrial calcium in the heart increases vulnerability to cardiac stress during the sleep-wake transition by dissipating membrane potential, slowing respiratory activities, and increasing ROS levels, and the change in stress vulnerability in the mitochondrial functions may explain the diurnal prevalence of cardiac pathologies [27].

Animal experiments have found that a high dietary calcium intake at dinner significantly increased serum TC, TG, and HDL-c levels, leading to lipid accumulation. This may be due to a high dietary calcium intake at night, leading to a lipid metabolism disorder due to a down-regulated clock [28]. Alterations in circulating lipids and ectopic lipid deposition impact the risk of developing CVD and metabolic diseases [29]. Therefore, dietary calcium may affect lipid metabolism through the regulation of circadian rhythm genes, resulting in adverse cardiovascular consequences.

It has also been found that the association of Δ with CVD risk was influenced by body weight. In the overweight population, the fifth quintile of Δ was significantly associated with CVD risk, suggesting that weight control may be a regulator of CVD prevention. The study found a significantly increased risk of early CVD death among the overweight population [30]. Overweight can also directly affect inflammatory factors and vascular homeostasis to increase cardiovascular risk, and it can also indirectly induce insulin resistance, T2DM, hypertension, and dyslipidemia to affect the occurrence and development of CVD [31].

This study has some advantages. First, this is the first study that used the largest nationally representative sample to assess the relationship between Δ and CVD in the United States adults. Second, this study highlighted that, under constant total calcium intake, dietary calcium intake should be properly distributed at breakfast and dinner. Third, our study adjusted for a series of potential confounding variables to improve the stability of the results. At the same time, we also recognize that this study has certain limitations. First, in observational studies, dietary recall methods are the most valid and commonly used instrument to collect dietary consumption information, and it is subject to lower measuring accuracy and efficiency, and there may be measurement errors. Second, although we adjusted for a wide range of major confounding factors during the analyses, the associations between Δ and CVD might be impacted by other unobserved and unknown confounding factors. Third, it is necessary to verify the conclusions through cohort studies in different races and countries.

## Conclusion

Compared to the lowest quintile of Δ, participants in the highest quintile of Δ were likely to experience CVD in the general population. It is, thus, necessary to scientifically allocate dietary calcium intake for breakfast and dinner.

## Data Availability

Data from NHANES was used in this study, which could be downloaded at www.cdc.gov/nchs/nhanes/index.htm.
